# Transposon Dynamics Drive Genome Evolution and Regulate Genetic Mechanisms of Agronomic Traits in Cotton

**DOI:** 10.3390/plants14162509

**Published:** 2025-08-12

**Authors:** Zeyu Dong, Shangkun Jin, Yupeng Hao, Ting Zhao, Haihong Shang, Zhiyuan Zhang, Lei Fang, Zhihong Zheng, Jun Li

**Affiliations:** 1 Hainan Institute of Zhejiang University, Sanya 572025, China; 2 Zhejiang Provincial Key Laboratory of Crop Genetic Resources, Key Laboratory of Plant Factory for Plant Factory Generation-Adding Breeding of Ministry of Agriculture and Rural Affairs, The Advanced Seed Institute, Zhejiang University, Hangzhou 310058, China; 3 Zhengzhou Research Base, State Key Laboratory of Cotton Biology, School of Agricultural Sciences, Zhengzhou University, Zhengzhou 450001, China; 4 Shihezi Academy of Agricultural Sciences, Shihezi 832000, China

**Keywords:** transposon, structural variation, *Gossypium genome*, agronomic trait, association analysis

## Abstract

Transposable elements (TEs) serve as important drivers mediating polyploidization events and phenotypic diversification in plant genomes. However, the dynamic changes in various TE subclasses post-polyploidization and their mechanisms of influencing phenotypic variation require further investigation. The allopolyploid *Gossypium* species, originating from two diploid progenitors, provide an ideal model for studying TE dynamics following polyploidization. This study investigated TE dynamics post-polyploidization based on 21 diploid and 7 polyploid cotton genomes. The Tekay subclass of the Gypsy serves as a major driver of *Gossypium* genome evolution, as it underwent two burst events in the At-subgenome and its progenitor, exhibiting the highest abundance, longest length, and largest proportion among all TE subclasses. In contrast, the Gopia superfamily Tork subclass has lower abundance but greater genic association, facilitating environmental adaptation and phenotypic variation. Additionally, a pan-TE-related structural variation, the pan-TRV map, was constructed by integrating resequencing data from 256 accessions. Genome-wide analysis of 28 cotton genomes identified 142,802 TRVs, among which 72,116 showed polymorphisms in the 256 *G. hirsutum* accessions. The Gypsy superfamily, particularly the Tekay subclass, has been identified as a major source of TRVs, while Copia-type elements demonstrate significantly greater enrichment in gene-proximal genomic regions. A total of 334 TRVs exhibiting statistically significant associations with 10 key phenotypic traits, including 164 TRVs affecting yield components and 170 TRVs determining fiber quality. This investigation delineates the evolutionary significance of transposable elements in *Gossypium* genome diversification while simultaneously providing novel functional markers and potential editing targets for genetic dissection and molecular breeding of key agronomic traits in cotton.

## 1. Introduction

Transposable elements (TEs) are widely distributed mobile genetic elements in genomes that replicate and integrate into new genomic locations through autonomous or non-autonomous mechanisms [1,2,3]. TEs are classified into two major groups based on their transposition mechanisms: Class I (retrotransposons) amplify via a “copy-and-paste” mechanism using RNA intermediates, while Class II (DNA transposons) transpose through a “cut-and-paste” DNA-mediated mechanism [4]. Class I elements constitute a substantial portion of plant genomes and can be further categorized into five orders: long terminal repeat (LTR) retrotransposons, DIRS (Dictyostelium intermediate repeat sequences), Penelope-like elements (PLEs), long interspersed nuclear elements (LINEs), and short interspersed nuclear elements (SINEs) [5,6]. Their classification is based on reverse transcriptase features, domain organization, and target site duplications (TSDs). Among these, LTR retrotransposons (LTR-RTs) dominate the genomes of major cultivated crops, such as rice [7], tomato [8], and soybean [9].

A defining feature of LTR-RTs is the presence of highly homologous long terminal repeats (LTRs) flanking coding regions [10,11], which include essential domains such as reverse transcriptase (RT), ribonuclease H (RH), integrase (INT), primer binding site (PBS), polypurine tract (PPT), and the Gag-Pol polyprotein gene [12]. LTR-RTs are primarily classified into two superfamilies, Copia and Gypsy [13]. Based on the arrangement of RT and INT in the Pol gene and sequence similarity, the Gypsy superfamily can be further subdivided into the Chromovirus, CRM, Tekay, and Athila subclasses, while Copia includes lineages such as Ivana, Osser, Bianca, and SIRE [4,14]. Multiple studies suggest that plant polyploidization events often coincide with bursts of TE amplification [15,16]. Following polyploidization, genome destabilization may impair TE suppression mechanisms, leading to TE activation [17,18]. LTR-RTs can cause genomic damage due to irregular insertions, prompting plants to evolve multilayered suppression mechanisms, such as silencing and purging mechanisms, to maintain genome stability [19,20,21]. The proximity of TEs to genes reflects the strength of these suppression mechanisms, indicating that TE insertions closer to genes indicate stronger purifying selection pressure. In crops, the efficiency of LTR-RT suppression mechanisms influences genome plasticity and domestication selection pressure [22,23]. Deciphering TE dynamics provides a molecular basis for improving stress resistance and genome stability in crop breeding.

Cotton (*Gossypium* spp.) serves as the most important natural fiber source globally, accounting for one-third of the annual demand [24]. Due to its unique genomic evolutionary characteristics, cotton has emerged as a model system for polyploidy research. The genus comprises 45 diploid species (2*n* = 26) encompassing eight genomic groups (A, B, C, D, E, F, G, K) and seven allotetraploid species (AD_1_–AD_7_, 2*n* = 52, AD genome) [24]. Its phylogenetic structure is strongly coupled with geographical distribution: the D and AD genomes occupy ecological niches in the Americas, the A, B, E, and F genomes are predominantly distributed across Africa and Asia, and Australian lineages (C, G, and K genomes) exhibit distinct island adaptations. This geographic–genomic correlation provides a natural experimental framework for investigating fiber development and polyploid adaptive evolution [25]. The current consensus suggests that the At-genome donor species of allotetraploid cotton A_0_ may be extinct, with this extinct diploid progenitor potentially serving as the common ancestor of *G. herbaceum* (A_1_) and *G. arboreum* (A_2_) [26]. Early studies identified A_1_ and A_2_ as putative At-subgenome donors [27,28,29]. Substantial evidence supports *G. raimondii* (D_5_) as the primary Dt-subgenome contributor to tetraploid cottons [26,30,31]. The rich genetic diversity of *Gossypium* species offers valuable resources for improving cultivated cotton. However, our current understanding of cotton genetics remains limited, as most genome-wide association studies (GWAS) focus solely on single-nucleotide polymorphisms (SNPs) and short indels [32,33], overlooking structural variations (SVs). As primary contributors to structural variations (SVs), transposable elements generate major-effect allelic variants through insertion polymorphisms that directly modulate transcriptional networks and shape phenotypic diversification in crops [34,35,36]. However, the specific contribution of TEs to cotton phenotypic variation remains unclear. In *Brassica oleracea*, TE insertions and point mutations independently activated functional genes, driving genome and phenotypic diversification in cabbage and its relatives [37]. Pan-genome analysis and population resequencing revealed significant associations between TE insertion polymorphisms and agronomic traits, including grain size and heading date in rice [38,39]. In *G*. *hirsutum*, Liu et al. systematically compared genomic features across 10 cotton species using the TM-1 genome as a reference. They identified 35,980 TRVs associated with 134,592 TEs and integrated these into a graphical genome. Through phenotypic *t*-tests and transcriptome analysis, this study uncovered lineage-specific TEs and identified key genes linked to cotton environmental adaptation and superior agronomic traits [40]. Although Liu et al. investigated trait-associated TEs using 10 genomes, their study did not account for the role of diploid cotton species in cultivated cotton improvement, and both marker and population sizes were limited.

The *Gossypium* genus, with its extensive genomic resources and evolutionary history, presents an ideal model for investigating TE dynamics after polyploidization and their role in agronomic trait variation [26]. Allotetraploid cottons originate from hybridization between two diploid progenitors, offering unique evolutionary insights. However, the post-polyploidization dynamics of TEs and their genetic impact on agronomic traits remain unclear. In this study, 28 genomes were analyzed, leading to the identification of 59 million full-length TEs, which were comprehensively classified into 16 well-defined subclasses. A systematic investigation was conducted on LTR retrotransposon (LTR-RT) distribution patterns among diploid cotton species, including length, abundance, subclass classification, and proliferation timing. Furthermore, post-polyploidization differences in TE distribution between diploids and tetraploids were thoroughly examined, covering subclass proportions, element length, and gene proximity relationships. This study aims to investigate the role of TEs in shaping genomes and phenotypic variation in cotton by developing a comprehensive TE-related variation (TRV) map. Furthermore, we seek to characterize key TE polymorphisms associated with agronomic traits, thereby expanding our understanding of TE-mediated genome evolution and its implications for crop improvement.

## 2. Results

### 2.1. Tekay Shapes the Genomes of Diploid Cotton Species

**Figure 1:**
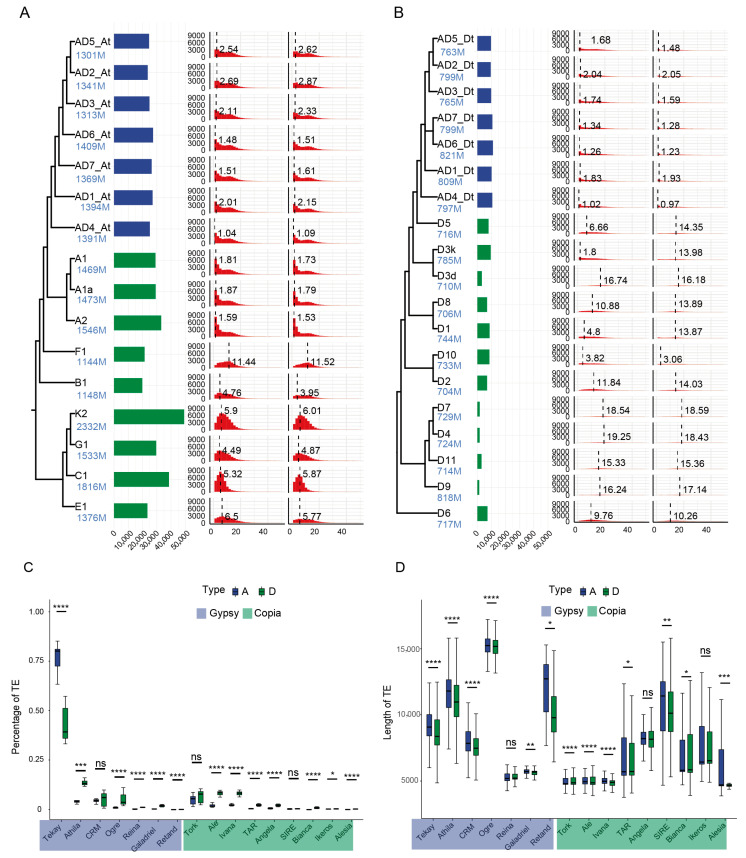
Identification and classification of full-length TEs across 28 Gossypium genomes. (**A**) Group A/At and (**B**) Group D/Dt- subgenomes. From left to right: phylogenetic relationships, TE abundance, burst timing, and Tekay expansion events (genome sizes in blue). (**C**) Comparison of transposable element subgroup proportions between A and D genomes in diploid cotton species. (**D**) Comparison of transposable element subgroup lengths across cotton species. Tekay and Retand are members of the Gypsy superfamily. Tork and Alesia are members of the Copia superfamily. Two-tailed *t*-test. **** *p* < 0.0001, *** *p* < 0.001, ** *p* < 0.01, * *p* < 0.05, ns *p* > 0.05.

A comprehensive analysis of full-length TEs was conducted across 28 *Gossypium* genomes, comprising 3 A-genome species, 12 D-genome species, 6 other diploid species, and 7 allotetraploid species (Figure 1, Figures S1A and S2A). These accessions were grouped into three categories based on evolutionary relationships, geographical distribution, and genome size: Group A (A-genome and other diploid cottons), Group D (D-genome cottons), and allotetraploids. A total of 591,107 intact TEs were identified (Figure 1A, Table S1), with Group D exhibiting the lowest variation (standard deviation: 1991), while Group A exhibited the most pronounced TE abundance variation (standard deviation: 9161). LTR-RTs were classified into Gypsy and Copia superfamilies. Gypsy elements were further divided into seven subclasses (Tekay, Athila, CRM, etc.), whereas Copia elements comprised nine subclasses (Tork, Ale, Ivana, etc.).

TE proliferation in Group A species occurred within 1.59-11.44 MYA (Figure 1A and Figure S1B), while Group D exhibited a wider range of recent TE activity within 1.8–19.25 MYA (Figure 1B and Figure S2B). The burst period in Group A species was notably later than in Group D. Analysis revealed congruent expansion patterns between Tekay-subclass and global TE bursts, suggesting that the proliferation of TEs was primarily driven by Tekay activation. Members of the A_1_-A_2_ clade in Group A underwent two distinct Tekay subclass expansion events. The first event coincided temporally with their sister lineage F1 (11.44 MYA), whereas the second event generated more abundant TE copies with more concentrated activation timing (Figure S1C). In contrast, Group D species showed only one Tekay subclass expansion event, characterized by an earlier burst timing and lower copy abundance (Figure S2C). The time of formation of tetraploid cotton during 1.10–2.72 MYA, comparative analysis with tetraploid subgenomes revealed that four out of seven in the At-subgenome displayed TEs burst events earlier than those in diploid ancestors (A_1_, A_1a_, and A_2_), suggesting polyploidization advanced the expansion timing of the At-subgenome. Conversely, TE activation in the Dt-subgenome occurred significantly later than in its diploid ancestor D5, implying that TE bursts in the Dt-subgenome may also have been directly driven by polyploidization.

A systematic quantification of 16 TE subclasses was performed across Group A, Group D, and allotetraploid Gossypium species. The Tekay subclass constituted the predominant component of full-length TEs (Figures S1D and S2D), exhibiting consistent dominance patterns with other Malvaceae species, which further confirms its critical role in Malvaceae genome evolution [41]. In Group A species, the Tekay proportion ranged from 66.14% to 85.18%, while in Group D species, it accounted for 33.12% to 57.20% (Figure S3, Table S2). Within allotetraploids, the At-subgenome maintained a stable Tekay proportion (73.33–76.56%), whereas the Dt-subgenome exhibited a lower but consistent proportion (36.63–42.99%) (Figure S4, Table S3). Within the Gypsy superfamily, the Tekay subclass was significantly more abundant in Group A than in Group D, while other TEs showed higher prevalence in Group D. Similarly, most Copia-class TEs exhibited significantly greater enrichment in Group D than in Group A (Figure 1C); the characteristic was also conserved between the At and Dt-subgenomes of tetraploid cotton (Figure S5, Table S4). Further, full-length TEs in Group A were significantly longer than those in Group D, consistent with the larger genome sizes observed in Group A species (Figure S6). Nearly all TE subclasses were longer in Group A compared to Group D (Figure 1D). This length divergence was maintained between the At- and Dt-subgenomes of allotetraploid cotton (Figure S7, Table S5). Within Group A and At-subgenome, an additional Tekay burst event, coupled with higher abundance, greater genomic proportion, and longer lengths of Tekay elements, demonstrates Tekay as key drivers of genomic diversification.

### 2.2. TE Dynamics Following Polyploidization in Cotton Species

**Figure 2:**
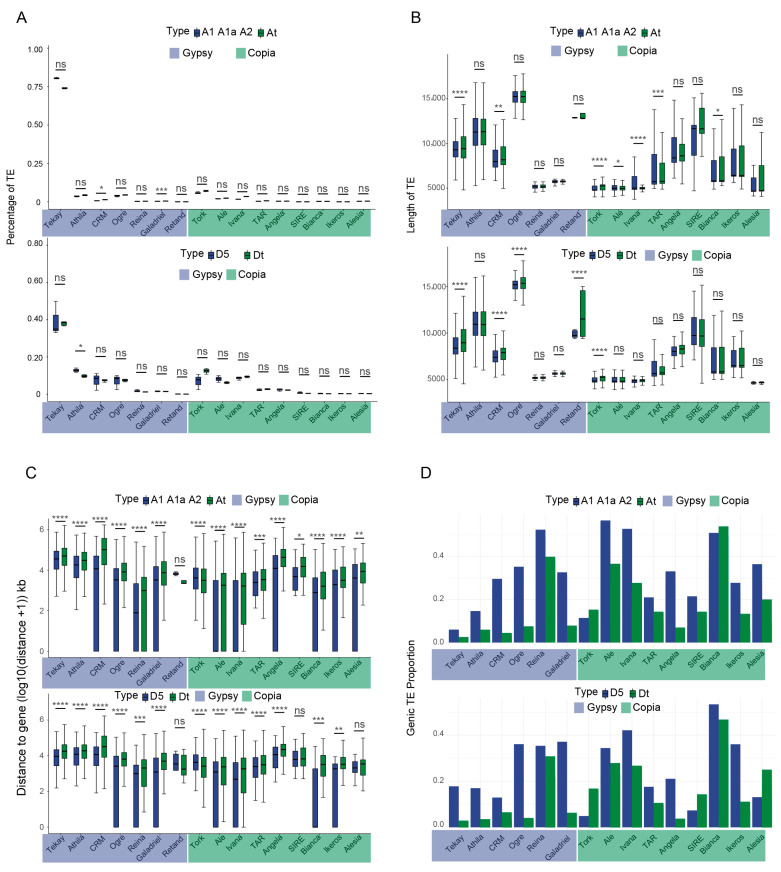
Evolutionary dynamics of TE subclasses in cotton polyploidization. Comparative analysis of TE subclass composition (**A**) and TE subclass length (**B**) between diploid progenitors and tetraploid subgenomes (At/Dt). (**C**) Physical distances between TEs and protein-coding genes in diploid progenitors and tetraploid subgenomes. (**D**) Differential enrichment of TE subclasses within genic regions following polyploid formation. Two-tailed *t*-test. **** *p* < 0.0001, *** *p* < 0.001, ** *p* < 0.01, * *p* < 0.05, ns *p* > 0.05.

This study conducted a comprehensive analysis of TE subclass proportions in tetraploid cotton and its diploid progenitors, revealing significant impacts of polyploidization on the distribution and dynamic changes of TE families. The At-genome is potentially derived from either A_1_ or A_2_, and the Dt-genome is derived from D_5_. A comprehensive analysis of TE subclass proportions in tetraploid cottons and their diploid progenitors was performed to elucidate the impact of polyploidization on TE dynamics. Post-polyploidization, Gypsy TEs exhibited an overall contraction trend in both At- and Dt-subgenomes (Figure 2A, Table S6). While most TE subclasses remained conserved, significant proportion changes were detected in specific subclasses. In the At-subgenome, significant expansion was observed for the CRM (*p* = 0.042), Galadriel (*p* = 0.00017), Ivana (*p* = 0.031), and TAR (*p* = 0.0021) subclasses. Conversely, the Athlia subclass displayed a significant reduction in the Dt-subgenome (*p* = 0.032). These findings strongly support the substantial influence of polyploidization on TE family distribution and dynamics.

Different TE classes exhibited distinct evolutionary trajectories for length variation following polyploidization (Table S7). The Tekay subclass showed significant length increase in both At- and Dt-subgenomes (*p* = 1.10 × 10^−72^ and *p* = 7.10 × 10^−35^, respectively). While the Ogre and Retand subclasses maintained stable lengths in the At-subgenome (*p* = 0.13 and *p* = 0.85, respectively), they displayed significant elongation in the Dt-subgenome (*p* = 3.20 × 10^−11^ and *p* = 8.90 × 10^−3^, respectively). In contrast, the Ale, Ivana, TAR, and Bianca subclasses showed significant length reduction in the At-subgenome (*p* = 2.10 × 10^−2^, 7.30 × 10^−11^, 5.80 × 10^−4^, and 3.70 × 10^−2^, respectively), but remained stable in the Dt-subgenome (*p* = 0.60, 0.69, 0.74, and 0.46, respectively) (Figure 2B, Table S7). Further, domain-specific length changes in retrotransposons during polyploidization were studied. For the At-subgenome, LTR regions (both 3′ and 5′) showed significant length reduction in tetraploids compared to diploid progenitors; all other protein-coding domains, except PROT, exhibited length expansion in tetraploids. For the Dt-subgenome, all examined domains demonstrated consistent length expansion in tetraploids (Figure S8). These findings demonstrate the diversified length dynamics of different TE subclasses post-polyploidization.

TEs located in genic regions and their flanking sequences may affect gene expression and potential function. A systematic comparison of TE–gene distances in cotton before and after polyploidization revealed significant impacts of polyploidization on TE distribution patterns and their proximity to genes. Following polyploidization, the distance between TEs and genes was generally increased (Figure 2C, Table S8). Post-polyploidization, TE–gene distances generally increased, with the Tekay subclass showing significantly greater distances in both the At (*p* < 2.2 × 10^−16^) and Dt-subgenomes (*p* = 2.00 × 10^−136^) (Figure 2C). We subsequently analyzed the distribution frequencies of different TE subclasses within genic regions. Most TE families showed decreased frequency in genes, including Gypsy subclasses (Tekay, Athlia, and CRM) and major Copia superfamily members (Ale, Ivana, and Angela). Notably, the Copia superfamily Tork subclass displayed increased retention in genic regions (Figure 2D) [42]. Polyploidization likely triggered genome restructuring and functional optimization, where defense mechanisms such as DNA methylation and chromatin remodeling suppressed or eliminated deleterious TEs near genes to reduce functional interference. Interestingly, Tork-like TEs may have been selectively retained due to their potential roles in evolutionary adaptation [43], possibly by modulating gene expression or contributing to adaptive evolution during polyploidization [15].

### 2.3. Construction of the TRV Genetic Map and Its Transcriptional Effects

**Figure 3:**
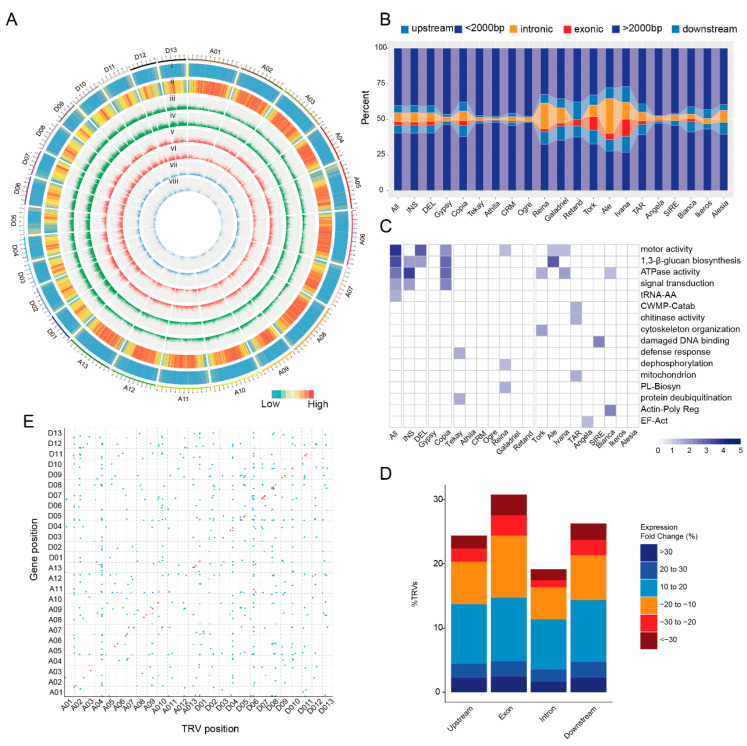
Landscape and transcriptional impact of TRVs. (**A**) Genomic distribution patterns across the 26 chromosomes of the TM-1 reference genome. (i) Gene density; (ii) TE annotation based on the reference genome; (iii) TRV distribution of insertion types; (iv) TRV distribution of deletion types; (v) Gypsy family; (vi) Copia family; (vii) Tekay subclass; and (viii) Tork subclass. (**B**) Distribution of TRVs over genic features. (**C**) GO-term analysis of genes with TRVs. (**D**) Proportion of TRV-containing genes with changes in transcription level in relation to the presence/absence of the TRV insertion. (**E**) Genome-wide distribution of significantly associated cis- and trans-eQTL loci (false discovery rate, FDR < 0.05) in *Gossypium hirsutum.* TRVs below the GWAS threshold are marked with red dots.

To investigate the impact of TEs on trait variation, genome-wide identification of TE-related variations (TRVs) was performed using genome assemblies of twenty-one diploid and seven tetraploid cotton species. A total of 312–15,318 TRV insertions were detected per genome, with an average of 5031.9 insertions (Figure 3A, Table S9). The G_1_ genome contained the fewest insertions, whereas AD_7_ exhibited the highest number. For deletion variations, 317–10,267 TRV deletions were identified per genome, averaging 4002.6 deletions, with G_1_ again showing the lowest and AD_7_ the highest counts (Table S9). Subsequently, 27 TRV variant sets were merged into a non-redundant dataset and mapped to the TM-1 reference genome, yielding 142,802 TRVs, including 67,638 insertions and 75,164 deletions. Furthermore, genotyping of 256 modern *G. hirsutum* accessions using this pan-genome framework detected 72,116 TRVs, comprising 29,126 insertions and 42,990 deletions. The relationship between genome number and marker quantity in *Gossypium* was systematically evaluated through 2000 random sampling replicates. Results revealed a decreasing trend in core TRVs and a continuous increase in pan TRVs with expanding genome numbers. Notably, incorporating diploid cotton species significantly enhanced TRV detection in cultivated *G. hirsutum* (Figure S9), providing a valuable marker resource for evolutionary and domestication studies. These findings significantly advance our understanding of TE-mediated structural variation in cotton genomics.

TRVs from distinct TE superfamilies exhibited distinct insertion patterns: Copia-derived TRVs preferentially occurred within or near genic regions, particularly in the Tork subclass, while Gypsy-related TRVs predominantly accumulated in intergenic regions (Figure 3B). Functional annotation revealed that genes harboring TRVs were significantly enriched in pathogen response and environmental stress-related pathways (Figure 3C). This functional bias was primarily driven by Copia insertions, reflecting their intrinsic targeting preference rather than purifying selection or detection artifacts. Experimental evidence from *Arabidopsis thaliana* and tomato (*Solanum lycopersicum*) further supported this conclusion [15,44], demonstrating conserved Copia insertion bias toward stress-responsive genes across divergent plant lineages.

A total of 80.74% of TRVs were located in intergenic regions, while 2.49% (1801 TRVs) resided within exonic regions, of which 43% (774 TRVs) induced frameshift mutations (Figure S10). To assess the impact of TRVs on gene expression in *G. hirsutum*, RNA-seq data from 196 accessions at 25 DPA fiber developmental stages were analyzed. All genes harboring TRVs within a 1 kb flanking region were examined, comparing transcript levels between accessions with and without insertions. Results demonstrated that TRVs in exonic regions exhibited a significantly higher proportion of genes with ≥10% expression change compared to other genomic regions (Figure 3D). A total of 75 cis-acting loci and 667 trans-acting loci were identified (Figure 3E). GO enrichment analysis revealed that these genes were significantly associated with lipid metabolic process, transporter activity, and defense response (Figure S11). These functional categories suggest potential roles in fiber quality improvement and environmental adaptation in *G. hirsutum*.

### 2.4. Identification of TRV-Associated Loci Contributing to Agronomic Traits in Cultivated Cotton

**Figure 4:**
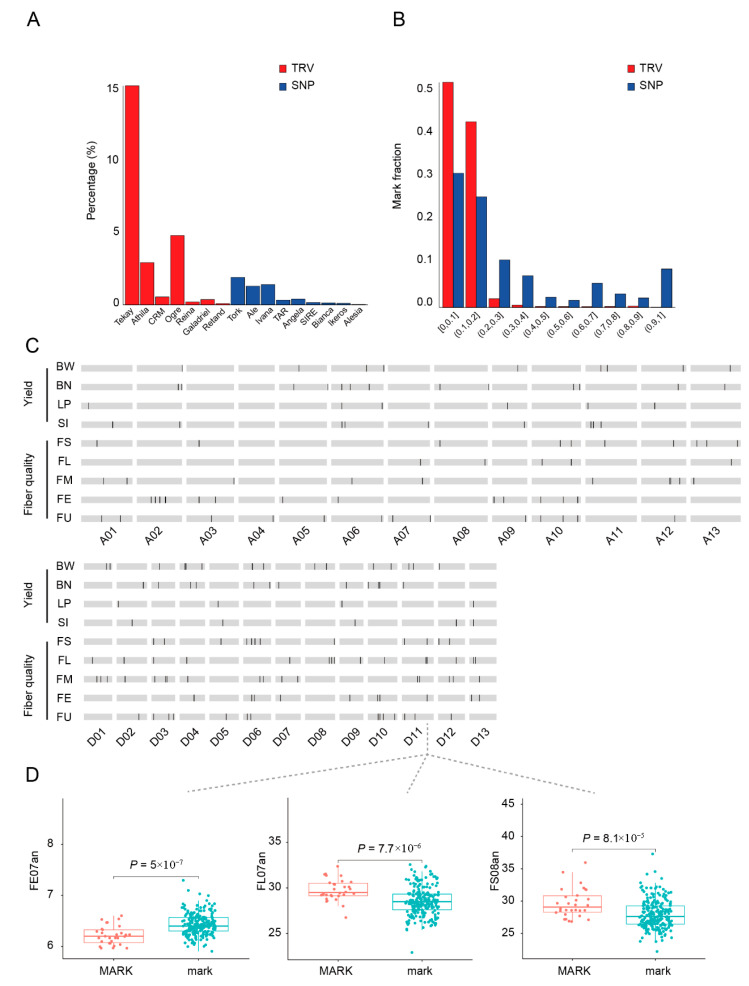
Identification of TRV features and GWAS analysis for yield and fiber quality traits in Gossypium hirsutum. (**A**) Number of detected TRVs per TE subclass. (**B**) Distribution frequency of TRV and SNP markers in the 256-population. The X-axis represents the proportion interval of population members, and the Y-axis represents the percentage of markers. (**C**) Distribution of trait-associated loci in cotton agronomic traits. In the chromosome diagram, trait-associated loci are marked by black vertical lines. (**D**) Comparison of fiber elongation rate, fiber length, and fiber strength traits based on the presence or absence of this TRV marker. Individuals with the mutation were designated as “Mark,” while those without the mutation were labeled “mark”.

Although the Tork subclass of Copia-class retrotransposons exhibited higher enrichment in genic regions, Tekay emerged as the dominant contributor to TRV markers, accounting for >15% of total TRV insertions (Figure 4A). To systematically evaluate whether TRVs could serve as a significant source of phenotypic variation, their population frequencies were compared with single nucleotide polymorphisms (SNPs). Results revealed that most TRVs occurred at low frequencies (<20% of accessions), mirroring the frequency distribution pattern of SNPs (Figure 4B). This research conducted comprehensive genome-wide association studies (GWAS) to investigate the genetic basis of nine agronomically significant traits in *G. hirsutum*. We analyzed multiple important cotton traits; this study focused on both fiber quality parameters (such as fiber length and strength) and yield-associated characteristics (including boll number per plant and lint percentage), providing valuable insights into the genetic architecture of these economically crucial features in upland cotton. The systematic GWAS approach enabled effective identification of genomic regions associated with these key agricultural traits. Comparison of SNP-GWAS and TRV-GWAS results demonstrated that TRV markers effectively complement SNP markers in identifying superior traits [36]. Notably, TRV-GWAS detected association signals absent in SNP-GWAS, increasing marker detection rates by 4.9–37.5% (Figure S12). GWAS was conducted using 72,116 TRV markers across five fiber quality traits and four yield-related traits, identifying 334 significantly associated markers. Among these, 288 were located in intergenic regions, while 46 were localized within or adjacent to genes (Figure 4C). Of the 46 genic/genic-proximal TRVs, 13 belonged to the Gypsy superfamily, and 11 were Copia superfamily members. Though full-length Gypsy elements accounted for 79.18% of total autonomous TEs, the difference in genic/genic-proximal insertions between the Gypsy and Copia superfamilies was only 15.3%, suggesting a genic insertion bias for Copia elements. A significant intergenic TRV marker (MARK) was identified on chromosome D11, associated with fiber elongation rate, length, and strength. Accessions carrying MARK (designated as MARK) showed significantly decreased fiber elongation but increased fiber length and strength compared to accessions without MARK (designated as mark) (Figure 4D). This finding demonstrates the insertion preference of Tork-class TEs in genic regions, providing a novel molecular marker resource for *G. hirsutum* genetic improvement while elucidating the potential role of TRVs in shaping key agronomic traits.

### 2.5. Key TRV Influencing Agronomic Traits in Cotton

TRV-GWAS was performed in *G. hirsutum* to analyze yield-related traits, including lint percentage, seed index, boll number, and boll weight. A total of 164 significant loci were identified, comprising 144 intergenic TRVs and 20 genic/genic-proximal TRVs (Table S11). Further, TRV-GWAS was conducted to analyze fiber quality traits, including length, strength, elongation, micronaire value, and fiber uniformity. In total, 170 significant loci were identified, comprising 144 intergenic TRVs and 26 genic/genic-proximal TRVs (Table S10).

**Figure 5:**
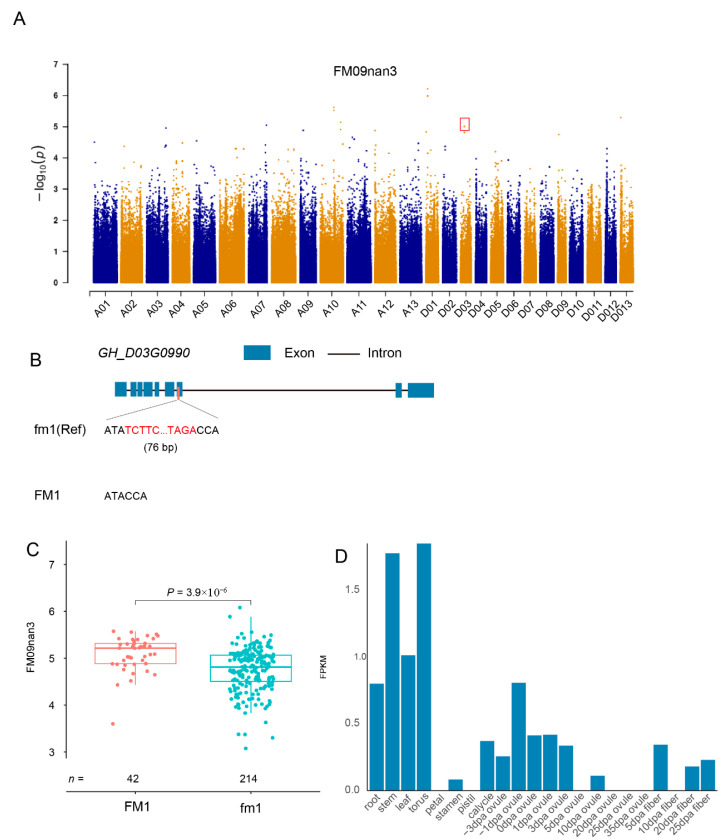
Identification of a TRV significantly associated with boll number. (**A**) Boll number-associated TRV GWAS. The TRV below the GWAS threshold are marked with red box. (**B**) A 319 bp insertion was identified 86 bp upstream of the *GH_D10G0066* promoter region. (**C**) Boll number comparison between accessions with/without the TRV marker. (**D**) Expression profiles of TRV-containing genes across tissues and during ovule/fiber development stages.

Boll number, defined as the number of effective bolls per plant, is a key agronomic trait for cotton yield. TRV-GWAS of boll number across 256 accessions identified 20 significant loci, including 9 insertions and 11 deletions, with an average length of 439.2 bp (range: 50–4632 bp). These variants were distributed as follows: thirteen intergenic, two gene regulatory, and five coding-region variations. A significant TRV (designated BN1/bn1) was detected on chromosome D10 (Figure 5A,B), located in the promoter region (−86 bp) of *GH_D10G0066*, encoding an α-amino-terminal protein methyltransferase (NTMT1_GOSHI). *GH_D10G0066* was found to regulate mitotic progression and DNA repair processes, directly influencing cell division activity in the fruiting branch meristem, thereby determining boll distribution and yield [45,46,47,48]. This variant potentially influences boll development via epigenetic regulation. Among 256 accessions, 72 (28.1%) carried BN1, which was associated with significantly reduced boll number compared to bn1 (Figure 5C). Additionally, *GH_D10G0066* exhibited expression in root and stem tissues (Figure 5D). Similarly, a micronaire-related variant (76 bp) was identified on chromosome D03 (Figure S13A,B) within exon 7 of *GH_D03G0990*, encoding a trehalase enzyme (TRE1_GOSHI). This enzyme hydrolyzes trehalose to glucose, potentially modulating carbon metabolism during fiber secondary cell wall thickening. *GH_D03G0990* potentially hydrolyzes trehalose to generate glucose, which may provide carbon and energy for secondary cell wall (SCW) biosynthesis during fiber development, consequently influencing fiber micronaire values [49,50]. In the 256-accession panel, 42 accessions (16.4%) carried this variant (Figure S13C), which was associated with significantly higher micronaire values. Expression analysis revealed temporal regulation of *GH_D03G0990* with peak expression at 5, 20, and 25 DPA (Figure S13D), coinciding with critical stages of fiber development.

Exonic variants can directly alter protein-coding sequences, leading to more pronounced phenotypic effects. This study systematically analyzed exonic TRVs for their influence on phenotypic variation and gene expression. Based on previously identified exon-spanning TRVs, population expression data were integrated to identify functional loci associated with superior traits. A total of 1801 exonic TRVs were identified. Through *t*-tests comparing expression levels and phenotypic data, 1143 variants (*p* < 0.05) showed significant phenotypic effects, while 38 (*p* < 0.05) induced expression level changes. Notably, 31 loci demonstrated concurrent associations with both gene expression and agronomic traits (Table S11). A key TRV on chromosome D05 was linked to fiber length, uniformity, strength, and micronaire value. This 208 bp insertion resided within exon 16 of *GH_D05G2599* (Figure S14A), encoding a 5′-3′ RNA exonuclease (XRN3 ortholog) involved in RNA metabolism and transcriptional regulation, an evolutionarily conserved function shared with Arabidopsis *AtXRN3*. The knockdown of *AtXRN3* in *Arabidopsis* resulted in altered expression of hundreds of genes accompanied by the accumulation of uncapped and polyadenylated read-through transcripts, which may interfere with normal expression of adjacent genes. In *G. hirsutum* fiber development, a similar mechanism could potentially lead to dysregulated expression of fiber-specific genes, thereby affecting fiber elongation and secondary cell wall biosynthesis [51,52]. Haplotypes were classified as MT1 (insertion-carrying) or mt1 (reference). Transcriptomic analysis revealed significantly lower *GH_D05G2599* expression in MT1 (Figure S14B). Phenotypically, MT1 accessions exhibited reduced fiber elongation but enhanced fiber length and strength (Figure S14C–E). Notably, this gene displayed ubiquitous expression across tissues (Figure S14F). These findings provide critical insights into the genetic basis of phenotypic variation and identify promising molecular markers for breeding applications in *G. hirsutum*.

## 3. Discussion

### 3.1. Dynamics of Lineage-Specific TEs During Polyploidization in Gossypium

Breakthroughs in cotton TE analysis were achieved in this study, which extends beyond previous classifications limited to category and superfamily levels. Through systematic classification and in-depth analysis of LTR retrotransposons in diploid and tetraploid cotton species, we revealed significant differences between A and D genome diploid cottons. Key comparisons included full-length TE abundance, length distribution, subclass proportions, and burst timing. Previous studies indicated that Gorge3 (*Gossypium* retrotransposable Gypsy-like element) underwent extensive proliferation in different-sized cotton lineages, significantly contributing to genome size variation [53,54]. However, the specific TEs subclass of Gorge3 remains uncharacterized, which we herein resolve. The results confirmed the Tekay subclass TEs influenced genome size and evolutionary divergence among different *Gossypium* species genomes through variations in their abundance, element count, insertion length, and amplification timing.

This research comprehensively analyzed full-length TEs across seven tetraploid cotton genomes, comparing At- and Dt- subgenomes with their putative ancestral species. Given the presumed extinction of the ancestral A_0_ genome, A_1_ and A_2_ genomes were used as proxies. By examining changes in TE content, subclass lengths, and TE–gene distances before and after polyploidization, we uncovered complex TE evolutionary patterns during genome doubling. TE–gene distance, an important indicator of purifying selection pressure, showed an overall increase post-polyploidization, particularly for Tekay subclasses in both At- and Dt- subgenomes. However, comparative analyses revealed Copia elements preferentially insert near or within gene-coding regions, potentially due to their structural features and transposition mechanisms, such as the Tork subclass. This distinct pattern suggests Copia elements may play important roles in species adaptation, leading to their evolutionary retention, a phenomenon corroborated by studies in tomato genomes [44,55]. In tomatoes, Copia elements not only show higher insertion frequency in genic regions but also maintain close associations with functional genes [44]. These findings provide critical evidence for understanding TE–host genome co-evolution while highlighting the potential regulatory roles of Copia elements in genome evolution and functional adaptation. The differential evolutionary trajectories of TE classes underscore their diverse impacts on genome architecture and phenotypic diversity in cotton species.

### 3.2. TEs Are a Major Source of Phenotypic Variation in Cultivated Gossypium Species

TRVs represent an important class of structural genomic variations that play a pivotal role in plant genome evolution and trait improvement. TRVs primarily comprise TE-mediated insertions and deletions, typically exceeding 50 bp in length, which are markedly longer than SNP and InDel markers. Studies have demonstrated the significant contribution of TE variations to trait enhancement in various species [36,38,44].

Using TM-1 as a reference, this study performed comprehensive genomic comparisons of 27 cotton species based on 591,107 full-length TEs, identifying 142,802 TRVs (67,638 insertions and 75,164 deletions). Compared to prior studies, our pan-TRV map substantially expanded the repertoire of genetic markers. Genotyping 256 resequenced accessions revealed 72,116 population-level TRVs (29,126 insertions, 42,990 deletions). TRV-GWAS analysis uncovered novel loci associated with TE variations and superior agronomic traits, demonstrating its utility as a complementary approach to SNP-GWAS for detecting cryptic genetic variations. The *GH_D10G0066* gene, encoding an alpha-amino-terminal protein methyltransferase (NTMT1_GOSHI), may affect boll number and yield through altered cell division efficiency caused by upstream TRV insertions [45,46,47,48]. A 76 bp exonic deletion in *GH_D03G0990*, encoding trehalase (TRE1), was significantly associated with elevated micronaire values [49,50]. Functional annotation suggested that TRE1 participates in carbon supply regulation during fiber secondary wall deposition by hydrolyzing trehalose into glucose. Expression profiling revealed stage-specific upregulation during critical fiber development phases (5, 20, and 25 DPA). In Arabidopsis, the ortholog *AtTRE1* regulates osmoregulation and sugar metabolism, while trehalose-6-phosphate synthase (TPS) in wheat modulates osmotic homeostasis and sucrose conversion [56]. Such osmotic adjustments may influence turgor-dependent cell wall expansion, thereby affecting secondary wall deposition patterns in fiber cells. The *GH_D05G2599* gene regulates transcript processing and accumulation, and TRV insertions within its coding region may disrupt gene function, causing cascading effects that influence various phenotypic traits [51,52], such as fiber elongation, which is positively regulated by genes such as *GhEXL3*, while aberrant RNA degradation mediated by *XRN3* may disrupt the transcript stability of these regulatory genes in *G. hirsutum* [57]. This pan-TRV study not only provides a novel analytical framework for cotton genomics but also serves as a reference for TE variation analysis in other crops. The pan-TRV map and TRV-GWAS methodology will facilitate deeper insights into the relationship between structural variations and phenotypic traits, offering new strategies for crop genetic improvement.

## 4. Materials and Methods

### 4.1. Published Genome and Transcriptome Data Collection

Complete genome assemblies of Malvaceae species were obtained from Phytozome, NCBI, COTTONOMICS [58], NGDC, and CottonGen databases [59]. A total of 28 genomes were retrieved, comprising 21 diploid and 7 allotetraploid cotton genomes (Table S12). For resequencing analysis, 256 accessions were downloaded under SRA accessions SRP047301 and PRJNA375965 (SRP106507). Corresponding transcriptome data from 196 samples were acquired under accession PRJNA1146873 [60].

### 4.2. Identification of Intact LTR

A comparative analysis of LTR-RTs was conducted using high-quality genomes of 28 cotton genomes. LTR-RTs were detected using LTR FINDER, LTRharvest, and LTRdigest [61,62]. In this analysis, we required that the distance between the two candidate LTRs be between 1 and 15 kb. Subsequently, the corresponding LTRs were identified, with lengths varying between 100 and 3000 bp and a similarity exceeding 80%.

### 4.3. Classification of Intact LTR Retrotransposons

LTR retrotransposon classification was performed using REXdb v3.0 [63] and LAST v983 [64], employing protein domain architecture analysis. Elements containing complete Gag-Pol sequences were classified as intact LTR-RTs (I) and further subdivided based on structural alignments. To validate Gypsy-class elements, we conducted tblastn alignments against the Gypsy database 2.0 [14], examining 3 kb flanking regions of LTR paralogs. Two additional categories were established: (1) Solo-LTRs (S) lacking Gag-Pol homologs, and (2) truncated LTR-RTs (T) retaining partial Gag-Pol homology.

### 4.4. Estimation of Insertion Time of the LTR-RTs

Upon genome integration of retrotransposons, their flanking LTR sequences typically exhibit high similarity. For insertion time estimation, only intact elements containing both LTRs were analyzed. LTR pairs were aligned using MAFFT (v7.221) with default parameters [65]. Divergence time (T) was calculated as T = K/2r, where K denotes LTR sequence divergence and r represents the Malvaceae-specific mutation rate, 2.6 × 10^−9^ [66].

### 4.5. Identification of Interspecific Transposon-Related Variations Among the Gossypium Genus

Using minimap2 (v2.24) with parameters “-ax asm5 --eqx” [67], we aligned 21 diploid and 6 allotetraploid Gossypium genomes to the TM-1 reference genome. Structural variations (SVs), particularly insertions and deletions (INDELs), were identified using SyRI [68], yielding 27 variant sets. To pinpoint TE-associated variations (TRVs), we extracted INDELs ≥ 50 bp and aligned these sequences against our previously annotated LTR-RTs using BLASTN (e-value ≤ 1 × 10^−5^, identity ≥ 90%). Variants with ≥90% sequence similarity to LTR-RTs were classified as TRVs.

### 4.6. Pan-TRV Construction

We constructed a pan-genome structural variation map for Gossypium species. First, non-redundant TRVs were obtained by merging 27 independent TRVs datasets using SURVIVOR (v1.0.7) with parameters: “merge sample_files 0.2 1 1 1 0 100” [69]. Subsequently, an TRVs-aware pan-genome (PanTRV) was constructed by integrating the merged TRVs into the TM-1 reference genome using the vg toolkit (v1.32.0) [66,70]. The genome graph was indexed with the automated workflow “vg autoindex -workflow giraffe -R XG”.

### 4.7. TRV Genotyping and Annotation

Whole-genome sequencing data from 256 accessions were uniformly aligned to the PanTRV reference graph, and population-level structural variant genotyping was performed using the ‘vg call’ command. For quality control, the population VCF files were filtered using BCFtools and VCFtools with stringent criteria: variants with missing call rates ≤ 30%, minor allele frequency (MAF) ≥ 0.05, and coverage depth ≥ 2× were retained to ensure analysis reliability. Functional annotation of TRV markers was conducted using ANNOVAR (https://annovar.openbioinformatics.org/en/latest/, 12 May 2025) [71,72,73].

### 4.8. Transcriptional Impact of TRV and eQTL Analysis

To examine the effects of TRVs on the expression of neighboring genes, RNA-Seq data from 25-day post-anthesis (DPA) cotton fibers were obtained from published studies. Gene expression levels were quantified as fragments per kilobase of exon model per million mapped reads (FPKM) using HISAT2 (v2.0.5) and StringTie (v2.1.7) based on the TM-1 genome annotation [74,75], and count normalization was performed using DESeq2 [76]. To assess the transcriptional regulatory effects of TRVs located in upstream, exon, intron, and downstream regions, comparative analyses of normalized transcript levels were conducted between TRV-carrying and non-carrying materials. In this population, TRVs exhibited three genotypes: complete absence (reference), heterozygous presence, and homozygous presence. The following two comparison schemes were applied: (1) absence vs. heterozygous presence and (2) absence vs. homozygous presence, where heterozygous and homozygous genotypes were treated as a single “presence” category in the first comparison. For phenotype–genotype association, statistical analyses were restricted to TRVs located in exonic regions, and *t*-tests were performed to compare transcript abundance differences between genotypes. Only genes exhibiting both significant phenotypic associations and transcriptomic variations were retained for further investigation.

In this study, we analyzed the relationship between TRV marks and gene expression abundance using 197 cotton germplasm accessions through the EMMAX association approach [77]. The transcriptome subset (*n* = 197 accessions) is a core collection nested within the 256-accession panel. The identified eTRVs were categorized into cis-eTRVs and trans-eTRVs according to their genomic positions relative to the associated gene’s transcription start/end sites, with a distance threshold of 1 Mb.

### 4.9. Genome-Wide Association Analysis Based on TRV

A genome-wide association study (GWAS) of the 72,116 TRVs identified in the 256-accession population was conducted using EMMAX [77]. Visualization of GWAS results was performed using CMplot (v4.4.1) [78].

## 5. Conclusions

This study systematically investigated the transposable element (TE) evolutionary patterns and their genomic structural impacts during polyploidization in Gossypium species. The Gypsy family’s Tekay clade played a pivotal role in diploid cotton genome evolution, with its abundant variation serving as the primary determinant of genome size diversity among cotton species. The Copia family’s Tork clade exhibited unique selection pressure patterns during polyploidization, demonstrating higher insertion frequency into genic regions.

Compared with traditional SNP/Indel markers, which only detect single-nucleotide or small-fragment variations, TE-related variation markers effectively capture large-fragment variations. TRV markers enhance the accuracy of GWAS, facilitate genomic selection, and provide valuable information for CRISPR target design and marker-assisted selection, thereby serving as a critical genetic resource for mining superior trait-related genes.

We identified 142,802 TE-related variants (TRVs) and conducted population genotyping analysis using 256 upland cotton accessions. Results revealed that 72,116 TRVs displayed polymorphism in cultivated cotton populations, with the Gypsy superfamily’s Tekay clade remaining the predominant contributor to genomic variation. Gene positional annotation showed that Tork elements had significantly higher insertion frequency within genic regions compared to other subgroups. Importantly, we identified several key genes regulating agronomic traits, such as a boll number-associated gene on chromosome D10. Although a set of key genes associated with structural variations was identified, functional validation remains to be performed. Further studies are required to verify candidate genes and elucidate the regulatory roles of structural variations. These findings not only elucidate the dynamic roles of TEs in cotton genome evolution but also provide valuable genetic resources and molecular markers for cotton molecular breeding.

## Data Availability

The original contributions presented in this study are included in the article. Further inquiries can be directed to the corresponding authors.

## Supplementary Materials

The following supporting information can be downloaded at: https://www.mdpi.com/article/10.3390/plants14162509/s1. Figure S1: Distribution Patterns of LTR Retrotransposons in Diploid (A-genome) and Tetraploid (At-subgenome) cotton. Figure S2: Distribution Patterns of LTR Retrotransposons in Diploid (D-genome) and Tetraploid (Dt-subgenome) Cotton. Figure S3: Proportions of Transposable Element Subgroups in Diploid Cotton Species. Figure S4: Proportions of Transposable Element Subgroups in Tetraploid Subgenomes. Figure S5: Comparison of Transposable Element Subgroup Proportions Between At and Dt subgenomes in Tetraploid Cotton Species. Figure S6: Comparison of Intact Transposable Element Lengths. Figure S7: Comparison of Transposable Element Subgroup Lengths Across At Dt genomes of Cotton. Figure S8: Domain Length Between Diploid Progenitors and Tetraploid Subgenomes (At/Dt). Figure S9: Number of Core/Dispensable TRV. Figure S10: Proportion Statistics of TRV Annotation Locations in the 256-Population. Figure S11: Go Enrichment Analysis of the eQTL Genes. Figure S12: Comparison of Association Mapping Results Based on TRV and SNP Markers. Figure S13: Identification of a TRV Significantly Associated with Fiber Micronaire. Figure S14: Effects of the TRV Marker on Multiple Phenotypic Traits. Table S1: LTR-RT Transposable Element Annotation Statistics Across 28 Cotton Species. Table S2: Subfamily Composition of Transposable Elements in Diploid Cotton Species. Table S3: Subfamily Distribution of Transposable Elements in Tetraploid Cotton Species. Table S4: Comparative Analysis of TE Composition Across Subtype. Table S5: Comparative Analysis of Transposable Element Length Across Subtype. Table S6: Dynamics of Transposable Element Composition Following Polyploidization. Table S7: Transposable Element Length Variation Pre- and Post-Polyploidization in Cotton. Table S8: Changes in TE Distance to Genes After Polyploidization. Table S9: Statistical Variation in TRV Copy Number and Size Among Genomes Relative to TM-1. Table S10: Summary of TRV-GWAS Results. Table S11: Summary of TRV-mediated Expression and Phenotypic Variations. Table S12: Genomic Characteristics of 28 Representative Cotton Species.
