# Supplementary material for: Transposon Dynamics Drive Genome Evolution and Regulate Genetic Mechanisms of Agronomic Traits in Cotton
Source: Plants (Basel). 2025 Aug 12;14(16):2509. doi: 10.3390/plants14162509 (PMC12389642; doi:10.3390/plants14162509)
Supplement: Supplementary file 1 [file plants-14-02509-s001.zip › SupFig_7_23.pdf]

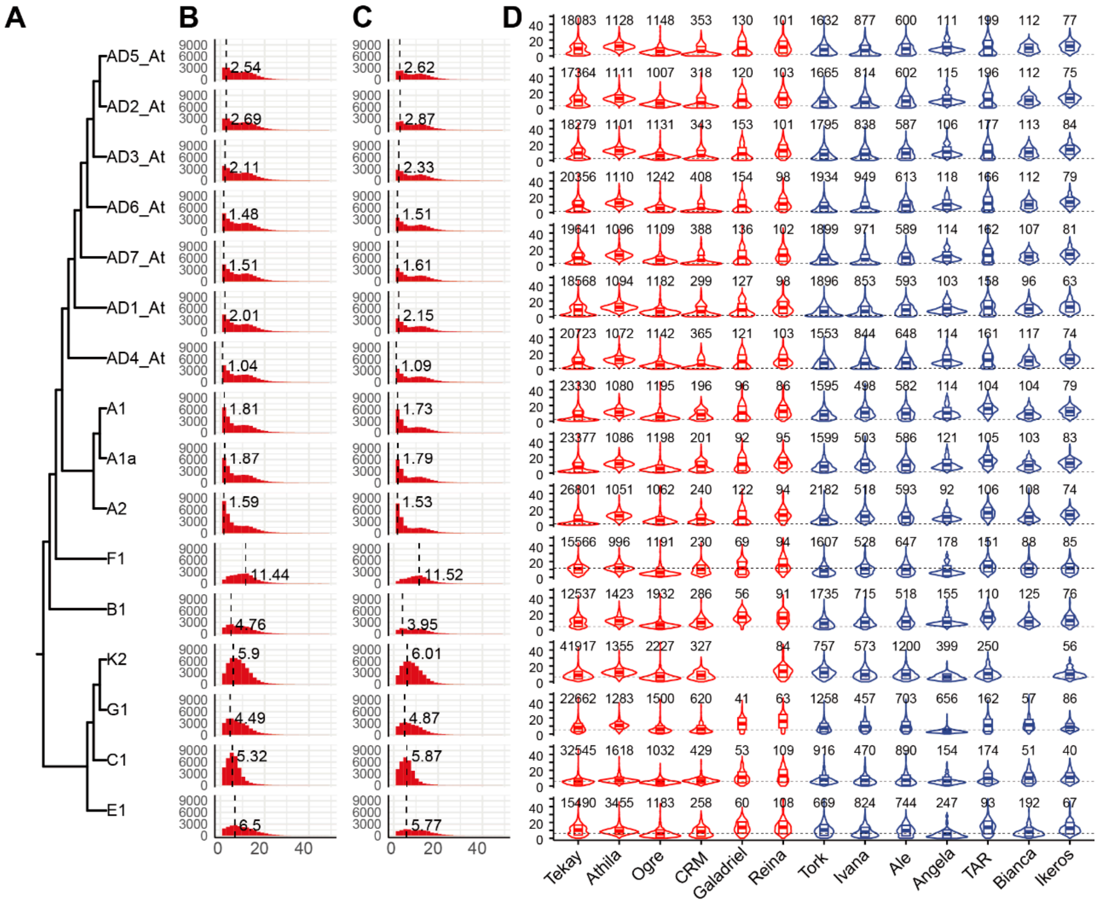

Figure S1 Distribution patterns of LTR retrotransposons in diploid (A-genome) and tetraploid (At-subgenome) cotton

(A) Phylogenetic relationships of the Malvaceae genomes are presented. (B) Distribution of insertion times of LTR-RTs in A group diploid and At cotton, with the black line indicating the peak period of LTR-RT bursts, and the Y-axis representing the number of burst LTR-RTs. (C) Distribution of insertion times of the Gypsy family in diploid cotton species, with the black line indicating the peak period of LTR-RT bursts, and the Y-axis representing the number of burst LTR-RTs. (D) Boxplots illustrate the distribution of burst events in transposable element subgroups, including only branches with more than 30 members. The numbers in brackets above the boxplots indicate the number of corresponding LTR-RT elements. The black dashed line corresponds to the peak period in B.

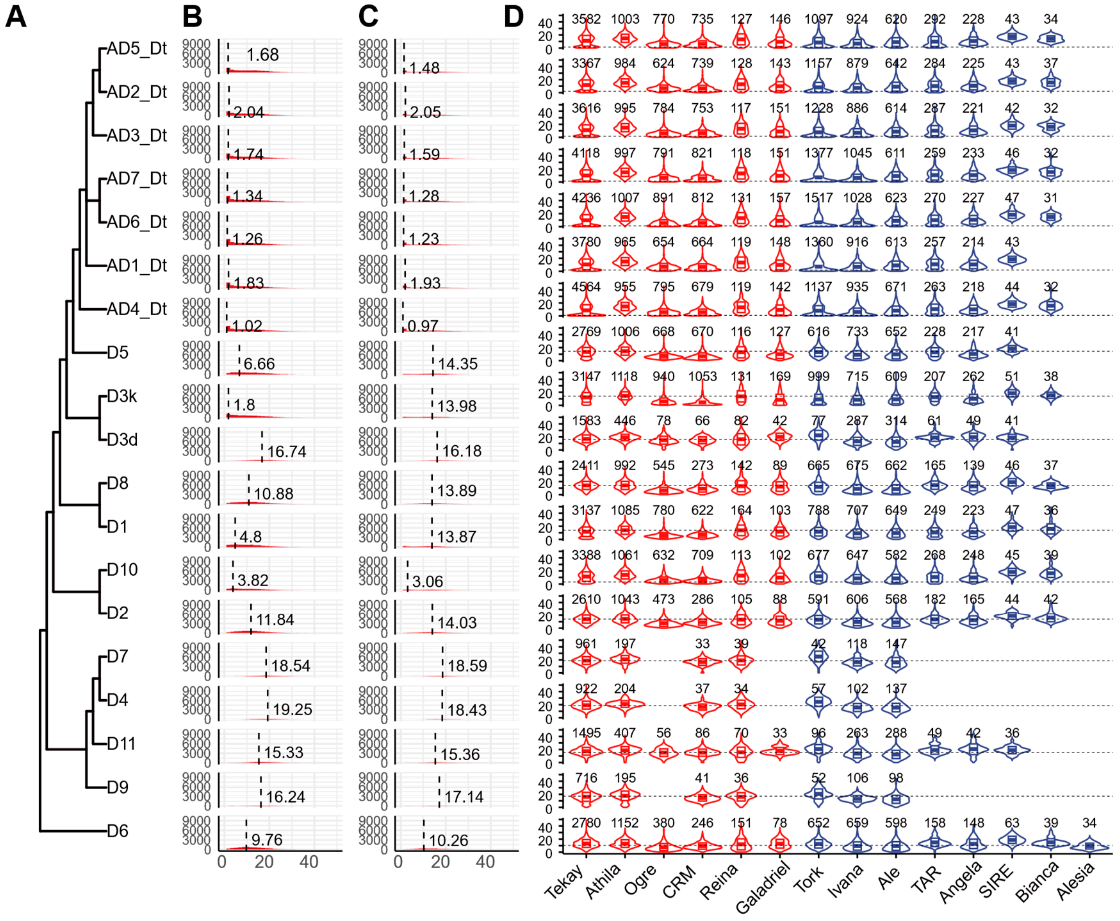

Figure S2 Distribution patterns of LTR retrotransposons in diploid (D-genome) and tetraploid (Dt-subgenome) cotton

(A) Phylogenetic relationships of the D-group diploid cotton species genomes are presented. (B) Distribution of insertion times of LTR-RTs in D-group diploid and Dt cotton species, with the black line indicating the peak period of LTR-RT bursts, and the Y-axis representing the number of burst LTR-RTs. (C) Distribution of insertion times of the Gypsy family in D-group diploid cotton species, with the black line indicating the peak period of LTR-RT bursts, and the Y-axis representing the number of burst LTR-RTs. (D) Boxplots illustrate the distribution of burst events in transposable element subgroups, including only branches with more than 30 members. The numbers in brackets above the boxplots indicate the number of corresponding LTR-RT elements. The black dashed line corresponds to the peak period in B.

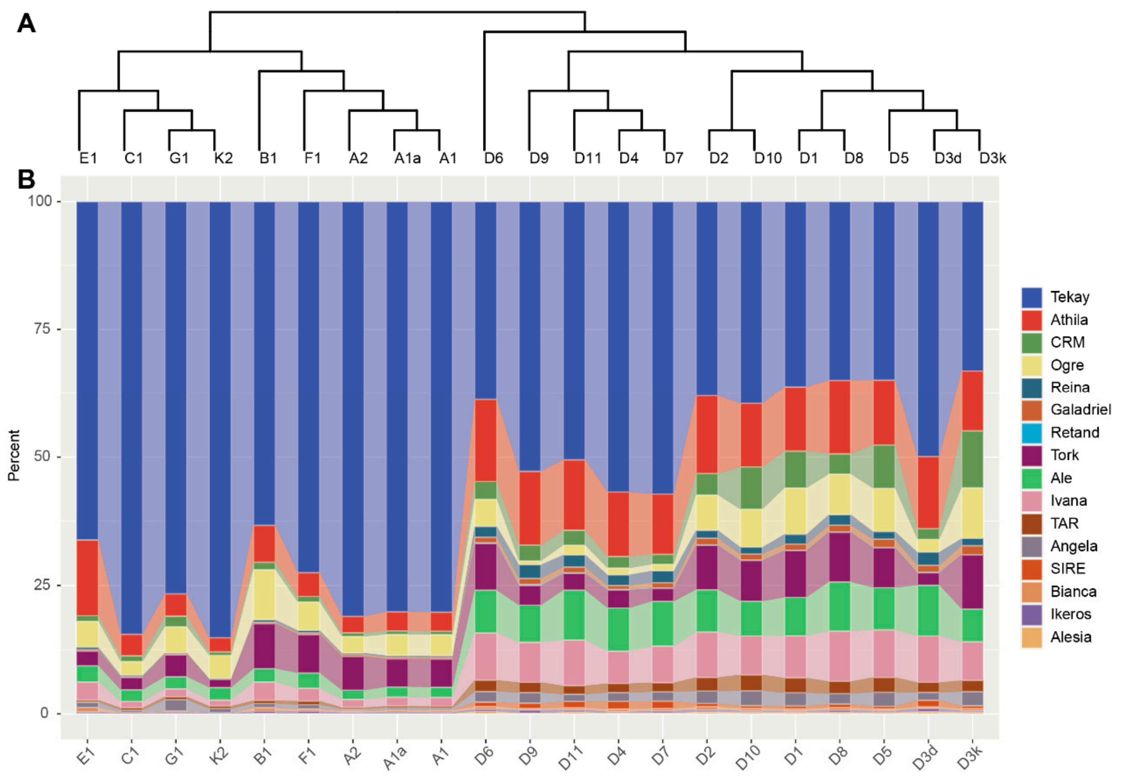

Figure S3 Proportions of Transposable Element Subgroups in Diploid Cotton Species

(A) Phylogenetic relationships of diploid cotton species. (B) Statistical analysis of transposable element subgroup proportions.

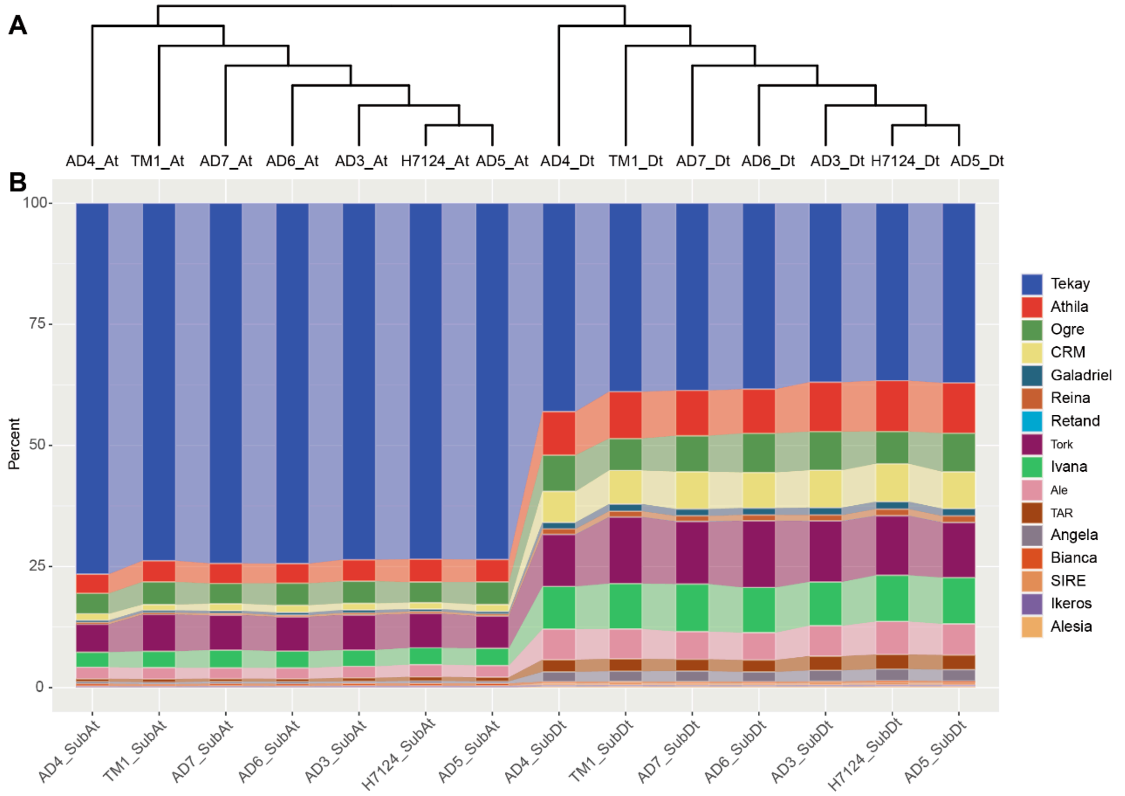

Figure S4 Proportions of Transposable Element Subgroups in Tetraploid Subgenomes

(A) Phylogenetic relationships of tetraploid subgenomes. (B) Statistical analysis of transposable element subgroup proportions.

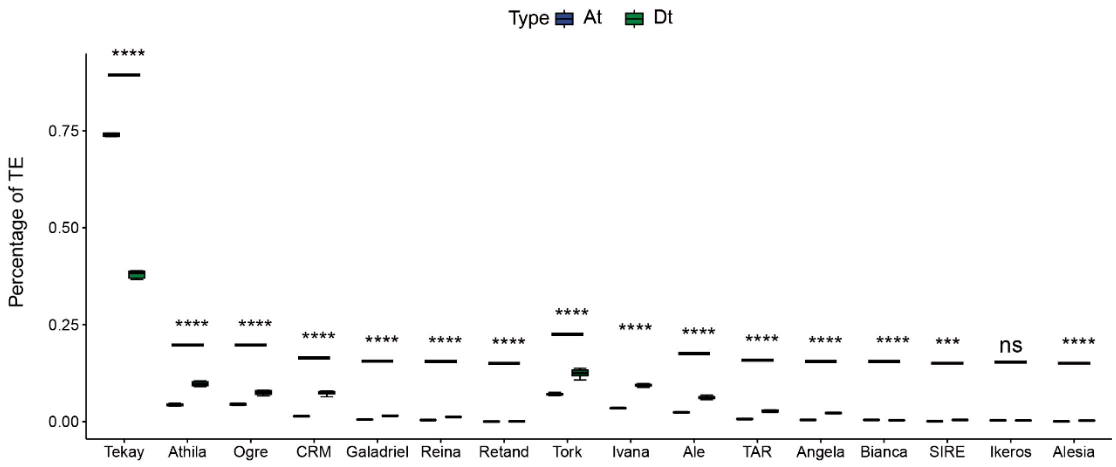

Figure S5 Comparison of transposable element subgroup proportions between At and Dt subgenomes in tetraploid cotton species

Tekay – Retand are members of the Gypsy supfamily. Tork – Alesia are members of the Copia supfamily. Two-tailed t-test. **** $P < 0.0001$, *** $P < 0.001$, ** $P < 0.01$, * $P < 0.05$, ns $P > 0.05$.

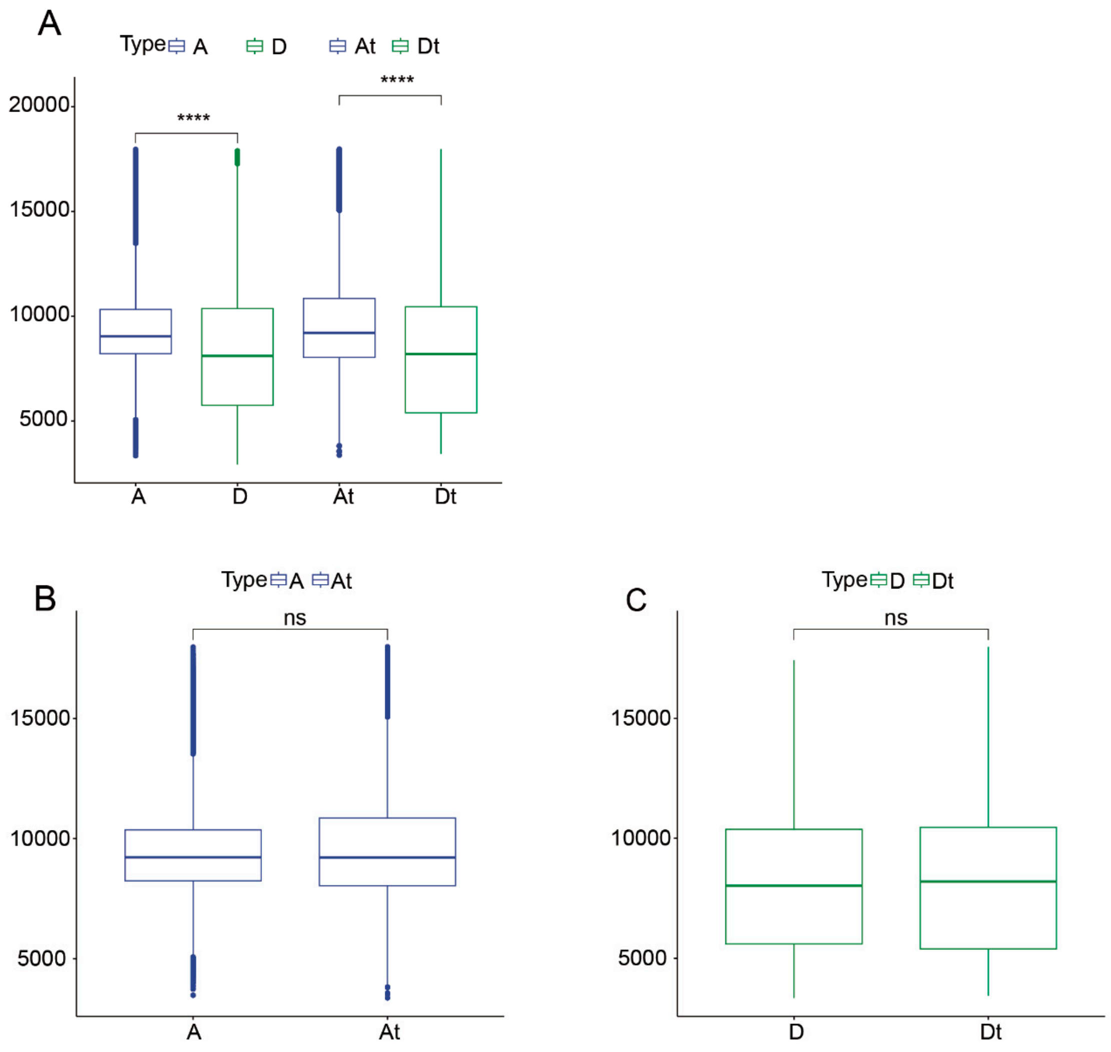

Figure S6 Comparison of Intact Transposable Element Lengths

(A) Boxplot of transposable element lengths based on A, D, At, and Dt genomes. (B) Boxplot of transposable element lengths based on A and At genomes. (C) Boxplot of transposable element lengths based on D and Dt genomes. Two-tailed t-test. **** $P < 0.0001$, *** $P < 0.001$, ** $P < 0.01$, * $P < 0.05$, ns $P > 0.05$。

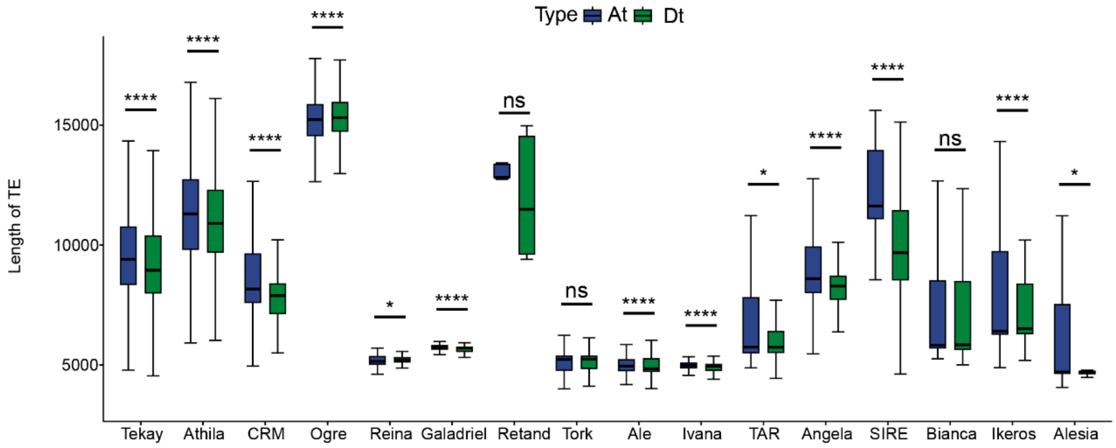

Figure S7 Comparison of Transposable Element Subgroup Lengths Across At Dt genomes of Cotton

Comparison of transposable element subgroup lengths between At and Dt subgenomes in tetraploid cotton species. Tekay – Retand are members of the Gypsy supfamily. Tork – Alesia are members of the Copia supfamily. Two-tailed t-test. **** $P < 0.0001$，*** $P < 0.001$，** $P < 0.01$，* $P < 0.05$，ns $P > 0.05$。

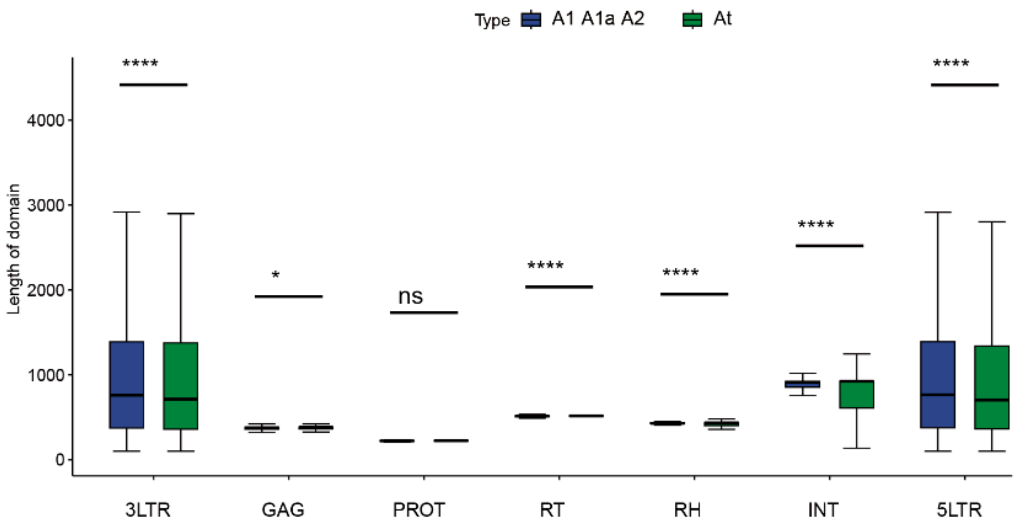

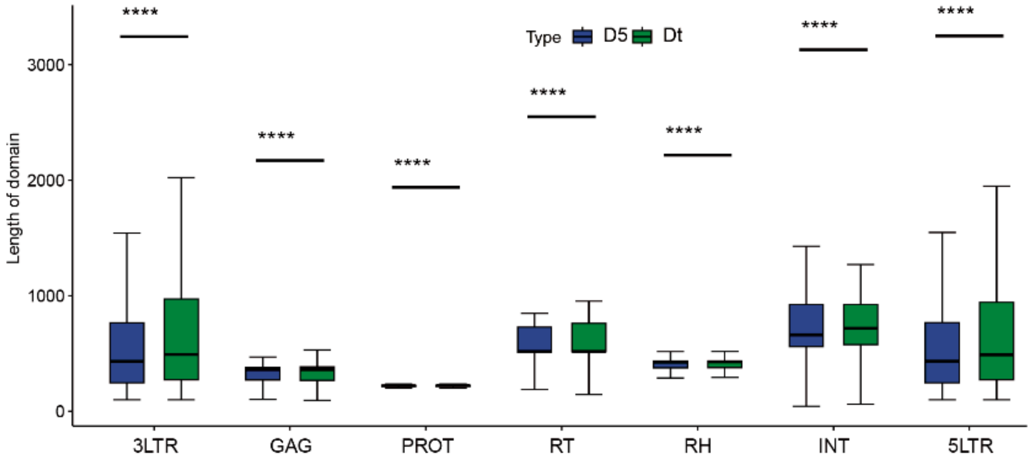

Figure S8 Domain Length between diploid progenitors and tetraploid subgenomes (At/Dt)

Tekay – Retand are members of the Gypsy supfamily.

Tork – Alesia are members of the Copia supfamily. Two-tailed t-test. **** $P < 0.0001$, *** $P < 0.001$, ** $P < 0.01$, * $P < 0.05$, ns $P > 0.05$。

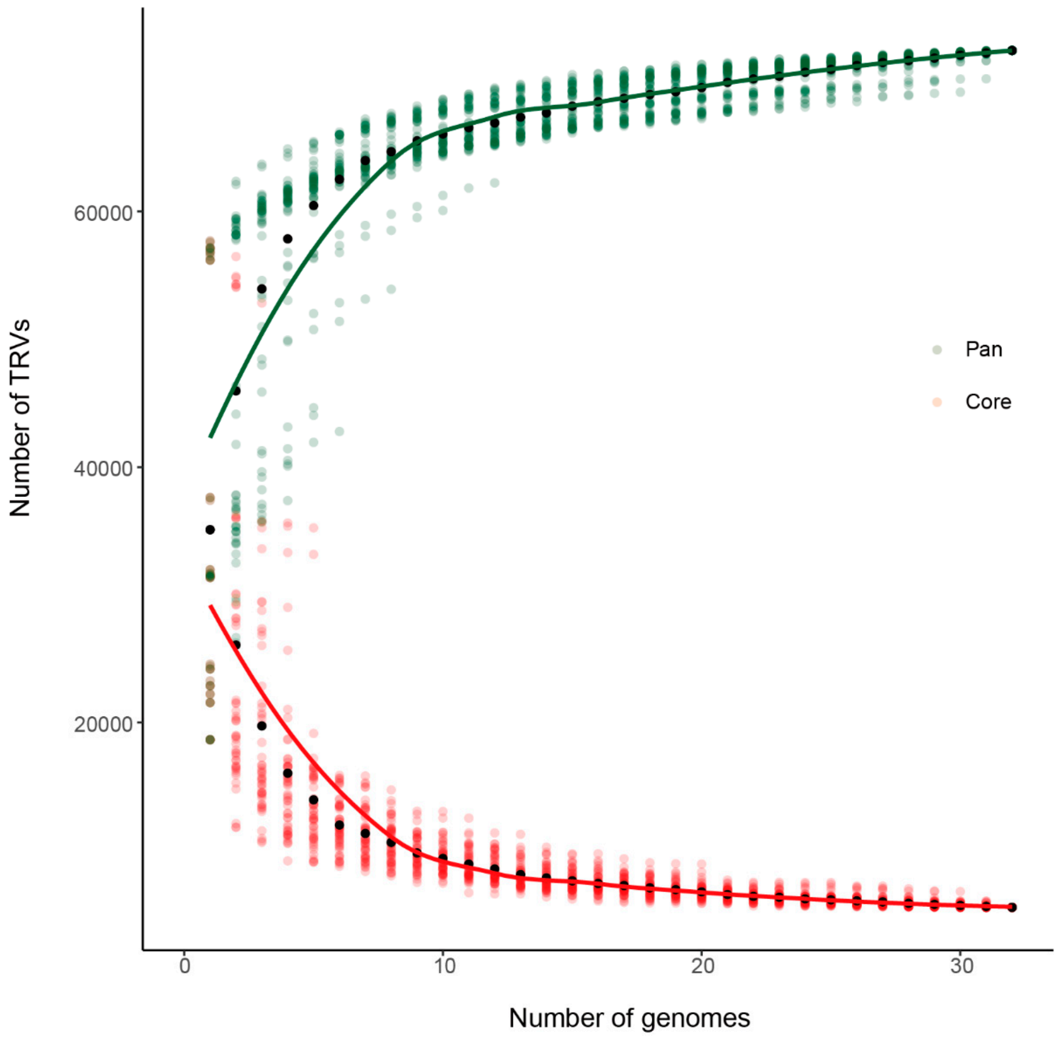

Figure S9 Number of Core/Dispensable TRV

Modeling analysis of the number of pan-TRV and core TRV in 256 cotton accessions. The upper and lower edges of the plot represent the 99% confidence interval.

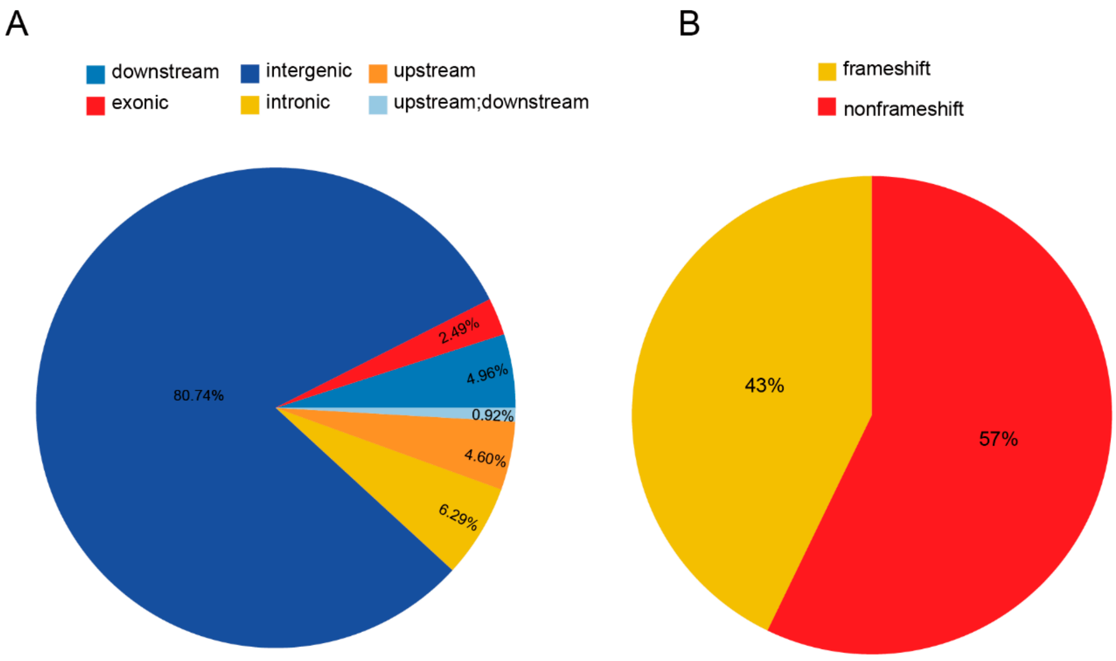

Figure S10 Proportion Statistics of TRV Annotation Locations in the 256-Population

(A) Distribution of TRV within the genome. (B) Proportion statistics of frameshift and non-frameshift mutations in exon regions.

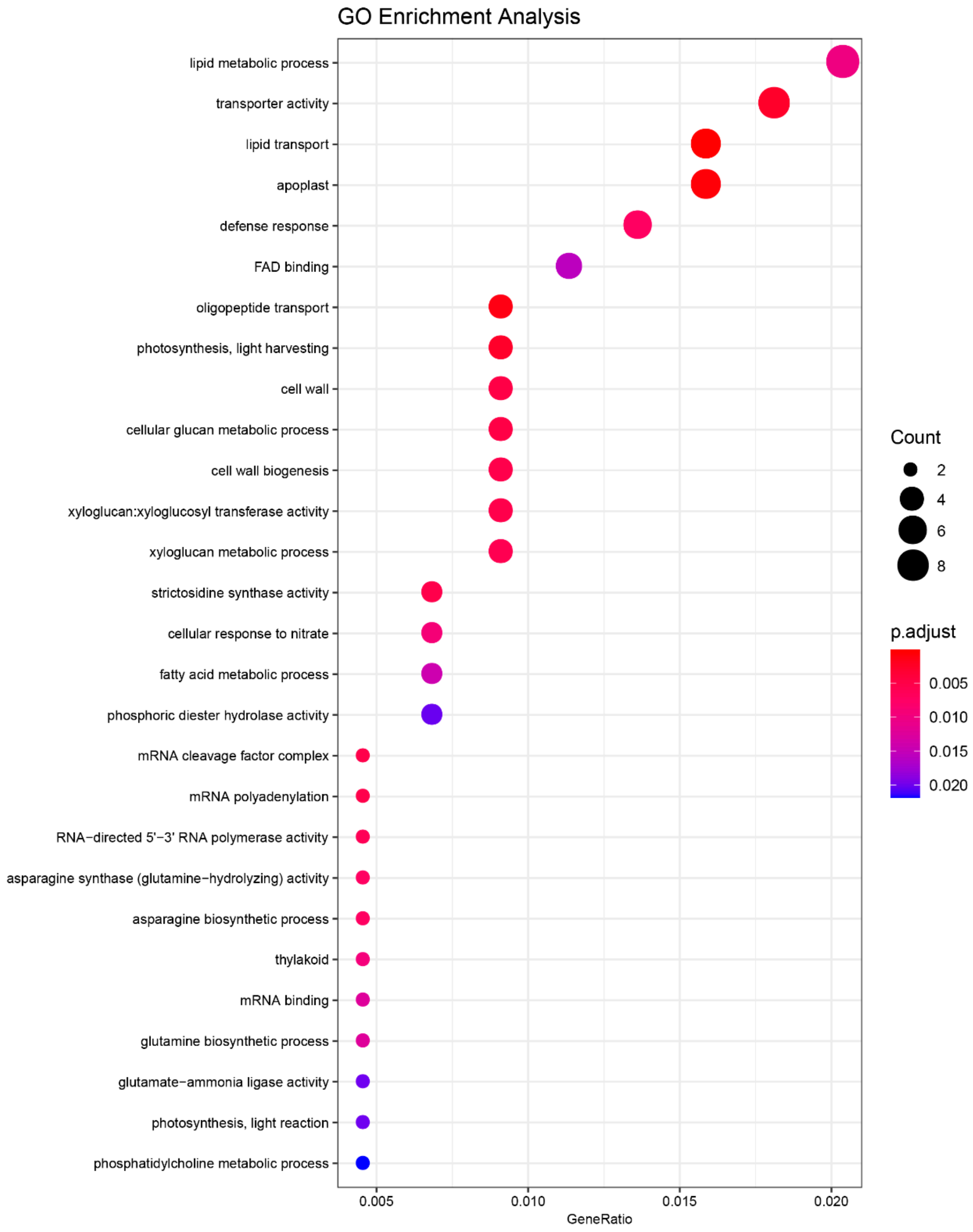

Figure S11 Go Enrichment Analysis of the eQTL genes.

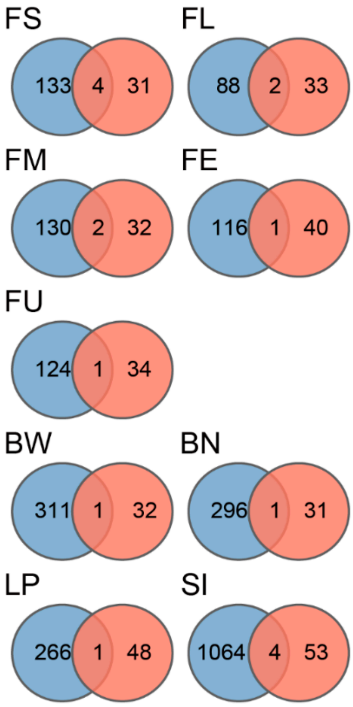

Figure S12 Comparison of association mapping results based on TRV and SNP markers. The number in the blue circle indicates the number of significant loci identified by SNP-GWAS. The number in the red circle indicates the number of significant loci identified by TRV-GWAS. The intersection represents the number of shared loci.

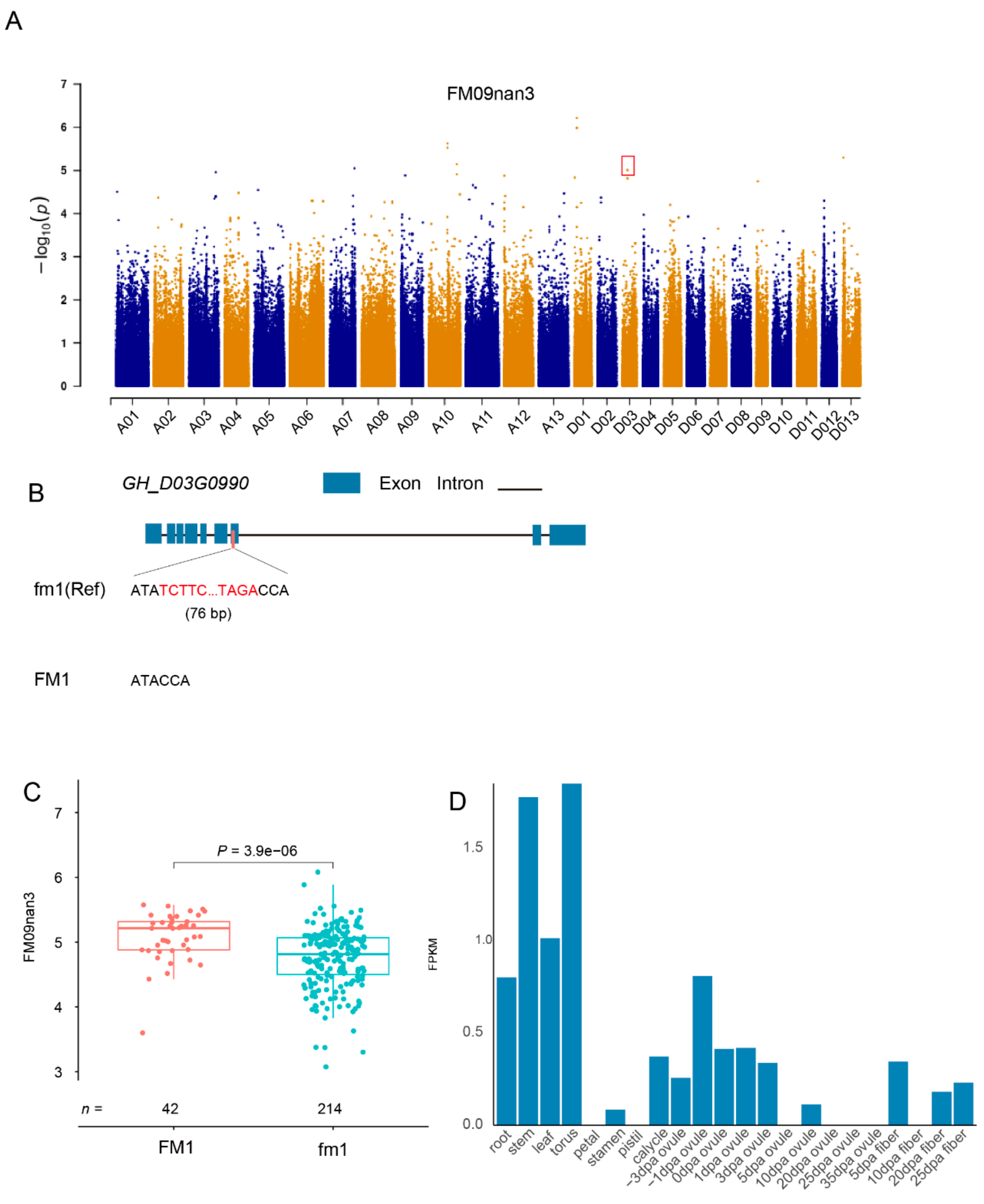

Figure S13 Identification of a TRV Significantly Associated with Fiber Micronaire

(A) TRV-based GWAS for fiber micronaire. (B) A 76 bp deletion was detected within the seventh exon of *GH_D03G090*. (C) Fiber micronaire comparison between cotton accessions with and without the TRV marker. (D) Expression profiles of the TRV-marked gene across tissues and ovule/fiber developmental stages.

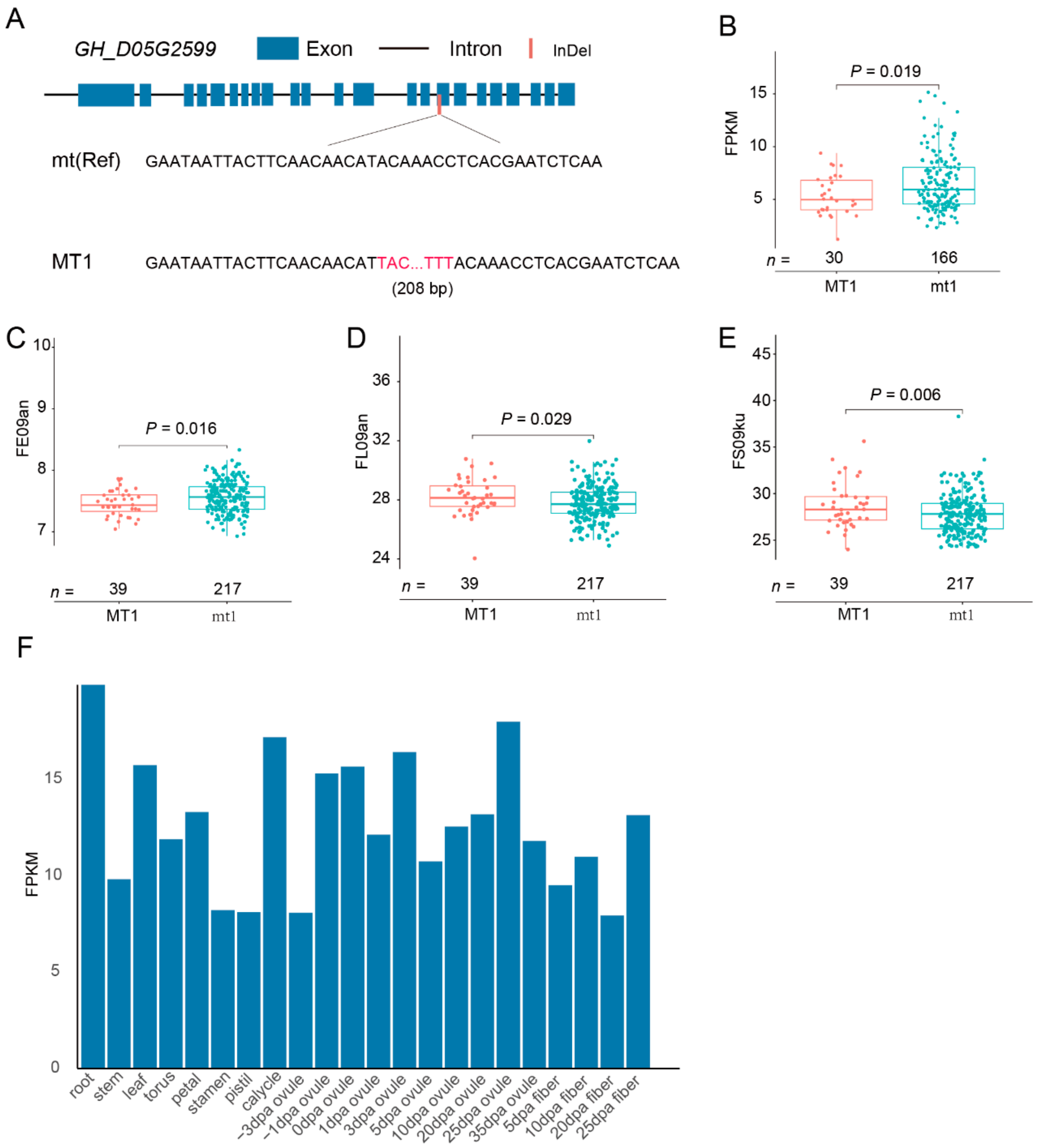

Figure S14 Effects of the TRV Marker on Multiple Phenotypic Traits

(A) A 208-bp insertion in the 16th exon of *GH_D05G2599*. (B) Box-plot analysis of gene expression differences in 196 germplasms grouped by presence/absence of the TRV marker. (C–F) Box-plot analysis of trait variations in 256 germplasms based on TRV marker status: fiber elongation (C), Fiber length (D), and uniformity (E). (F) Expression profiles of the TRV-marked gene across tissues and ovule/fiber developmental stages.
